# Effects of Alkaline Hydrogen Peroxide and Cellulase Modifications on the Physicochemical and Functional Properties of *Forsythia suspensa* Dietary Fiber

**DOI:** 10.3390/molecules28207164

**Published:** 2023-10-19

**Authors:** Kejing Yan, Jiale Liu, Wensheng Yan, Qing Wang, Yanxiong Huo, Saisai Feng, Liangliang Zhang, Qingping Hu, Jianguo Xu

**Affiliations:** 1 College of Food Science, Shanxi Normal University, Taiyuan 030031, China; 18835735152@163.com (K.Y.); 16635711350@163.com (J.L.); yws9607@163.com (W.Y.); huoyanxiong195615@163.com (Y.H.); fengsaisai@outlook.com (S.F.); uc8811@126.com (L.Z.); 2 College of Life Science, Shanxi Normal University, Taiyuan 030031, China; wq010501@163.com

**Keywords:** dietary fiber, *Forsythia suspensa*, modification, physicochemical property, functional property

## Abstract

Besides active substances, *Forsythia suspensa* is rich in dietary fiber (DF), but it is often wasted or discarded and not put to good use. In order to improve the function of *Forsythia* DF, it was modified using alkaline hydrogen peroxide (AHP) and cellulase (EM). Compared to the control DF (ODF), the DF modified using AHP (AHDF) and EM (EMDF) had a looser microstructure, lower crystallinity, and higher oil holding capacity (OHC) and cation exchange capacity (CEC). The AHP treatment significantly increased the water holding capacity (WHC) and water swelling ability (WSA) of the DF, while the EM treatment achieved just the opposite. Moreover, the functional properties of AHDF and EMDF, including their cholesterol adsorption capacity (CAC), nitrite ion adsorption capacity (NAC), glucose adsorption capacity (GAC), glucose dialysis retardation index (GDRI), α-amylase inhibitory activity, and DPPH radical scavenging activity, were far better than those of ODF. Together, the results revealed that AHP and EM modifications could effectively improve or enhance the physicochemical and functional properties of *Forsythia suspensa* DF.

## 1. Introduction

Dietary fibers (DFs) can be defined as health-beneficial carbohydrate polymers that resist digestion by small intestinal enzymes [1]. Given the increasing evidence suggesting its benefits on health, DF is drawing more and more interest among researchers [2]. DFs, as complex and heterogeneous organic polymers, are mainly composed of monosaccharides that are linked by a variety of glycosidic bonds and intertwined with other phytochemical compounds, such as polyphenols, flavonoids, and terpenoids. A large amount of functional groups, including aldehydes, carboxylic acid, and hydroxyl groups, shape not only the unique secondary or high-level structures of these polymers, but also their specific functional characteristics, such as their abilities to reduce the absorption of macronutrients (carbohydrates, fats, and proteins), inactivate free radicals, and regulate the host immune response [3,4]. Therefore, some manufacturers supplement food with dietary fibers through exogenous addition in order to increase its health benefits. Jiang et al. reported that the proper addition of insoluble dietary fiber from ginseng residue improved the nutritional components, polyphenolic compounds, antioxidant activities, and sensory scores, as well as the NO_2_^−^ ion, cholesterol, and bile acid adsorption capacities, of supplemented bread samples [5]. The DF from kinnow peels (*Citrus reticulata*) has been used to develop cookies that showed good nutritional, sensory, and textural properties, with an overall acceptability of 7.15–7.84 [6]. Furthermore, DF is also used in beverage production, improving beverages’ sensory characteristics and their relationship with consumers’ liking, wanting, and sensory satisfaction [7]. Modifying DF to satisfy the demands of consumers or to increase its yield has gained a lot of attention in the field of functional food development and processing.

Currently, physical, chemical, biological, or combined modification methods have been applied to change the physiochemical properties of DFs or improve their function [8]. Hydrogen peroxide is an efficient low-toxicity and low-cost fungicide, and it is widely used in industrial bleaching and surgical disinfection, as well as in other industries. Alkaline hydrogen peroxide (AHP) is used in the pretreatment of lignocellulosic biomass to reduce its resistance to enzymatic hydrolysis and improve its bioavailability [9]. AHP modification is one of the commonly used chemical modification methods with great economic advantages that leads to partially degraded, depolymerized, and fragmented cellulose, hemicellulose, and lignin [10]. More importantly, AHP treatment of biomaterials leads to the formation of oxygen and water as the reaction products, without causing secondary pollution. Many studies have shown that AHP modification could improve the solubility of DFs, increase their binding capacity to fats or glucose, and strengthen their water holding capacity (WHC) and functional properties [10,11]. Cellulase, an enzyme that catalyzes cellulose hydrolysis, provides an ecofriendly biomodification means for DF [12]. Through the fragmentation effect of cellulase, modified DFs exhibited an increase in oil holding capacity (OHC), WHC, and cholesterol absorption capacity (CAC) [13,14].

*Forsythia suspensa* is a herb widely planted in China, which has some medicinal value for treating diseases such as diabetes, acute nephritis, and obesity [15]. In addition, *Forsythia* leaves have been used as tea for hundreds of years among Chinese people [16,17] and were listed in the homologous catalogue of medicines and food of the National Health and Family Planning Commission in 2018. *Forsythia* flowers can also be processed into tea or natural plant pigments used as food additives [17,18]. *Forsythia suspensa* can be made into tea for drinking purposes. A certain amount of *Forsythia suspensa* can be added to boiling water: the decocted juice will brew green tea that can be drunk. Alternatively, *Forsythia suspensa* and green tea can be drunk directly with boiling water. *Forsythia suspensa* can also be drunk with green peels, trichosanthis, peach kernels, and orange leaves. In addition, processed *Forsythia suspensa* is also a promising DF resource due to its low cost [19] and high content of fiber [20]. However, the poor physicochemical properties and low soluble DF concentration of *Forsythia suspensa* mean that its DF is greatly limited in its application in foods [21]. Unfortunately, no studies on the utilization and modification of *Forsythia suspensa* DF have been reported yet. It is uncertain whether the physicochemical and functional properties of *Forsythia suspensa* DF could be improved after AHP and EM modifications.

In the current study, the effects of two common modification means, namely AHP and EM treatments, on the structural, physicochemical, and functional properties of DF from *Forsythia suspensa* were evaluated to provide theoretical support for the modification and application of *Forsythia suspensa* DF.

## 2. Result and Discussion

### 2.1. Color Analysis

The colors of the samples are presented in Table 1. For EMDF and AHDF, the values of a* and b* were lower than those of ODF, which means that the EMDF and AHDF samples were greener and bluer than the ODF sample. Similarly, the values of a* and b* in the DF from purple turnips were reduced after AHP and EM modifications [22]. These phenomena could be attributable to the oxidation effect of H_2_O_2_ and the hydrolysis of cellulase [22], which causes a change in the structure of the chromogenic substance of *Forsythia suspensa* DF. The ΔE values implied that the colors were different before and after the treatments. Thus, these results indicated that the AHP and EM modifications changed the color of the DF. However, concerning the color of the DF, there was no remarkable difference between the AHP and EM modifications.

### 2.2. Structural Analysis

#### 2.2.1. SEM Analysis

**Figure 1:**
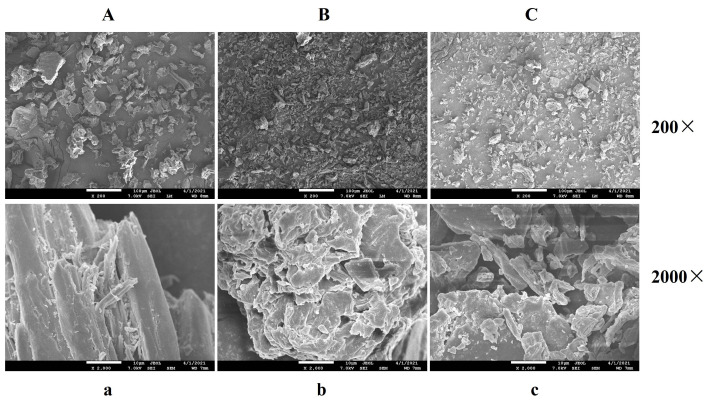
Scanning electron microscopy images of ODF (**A**,**a**), AHDF (**B**,**b**), and EMDF (**C**,**c**).

The microstructure of the DF samples was observed via SEM (Figure 1). When compared with the ODF sample, it was found that the surface structure of the DF showed significant differences after the AHP and EM treatments. The surface of ODF was flat and compact (Figure 1A,a), while that of AHDF seemed to have a honeycomb-like structure, consisting of many holes and folds (Figure 1B,b). These findings were similar to the results found in okara after AHP modification [23]. This surface wrinkling of AHDF could be due to the breakage of the fiber macromolecules [24]. Meanwhile, the surface structure of EMDF was looser and more porous when compared with that of ODF (Figure 1C,c). After the EM treatment, the glycosidic linkages of polysaccharide chains in the DF were partially disrupted [25]. This might be the reason why some loose and porous structures were present on the surface of EMDF. Moreover, these structures could increase the surface area of the DF, as well as exposing some active groups, thus providing abundant sites for facilitating the interaction between the DF and other components [2]. Therefore, these observations indicated that the biological activity of the modified *Forsythia suspensa* DF was improved.

#### 2.2.2. TGA Analysis

**Figure 2:**
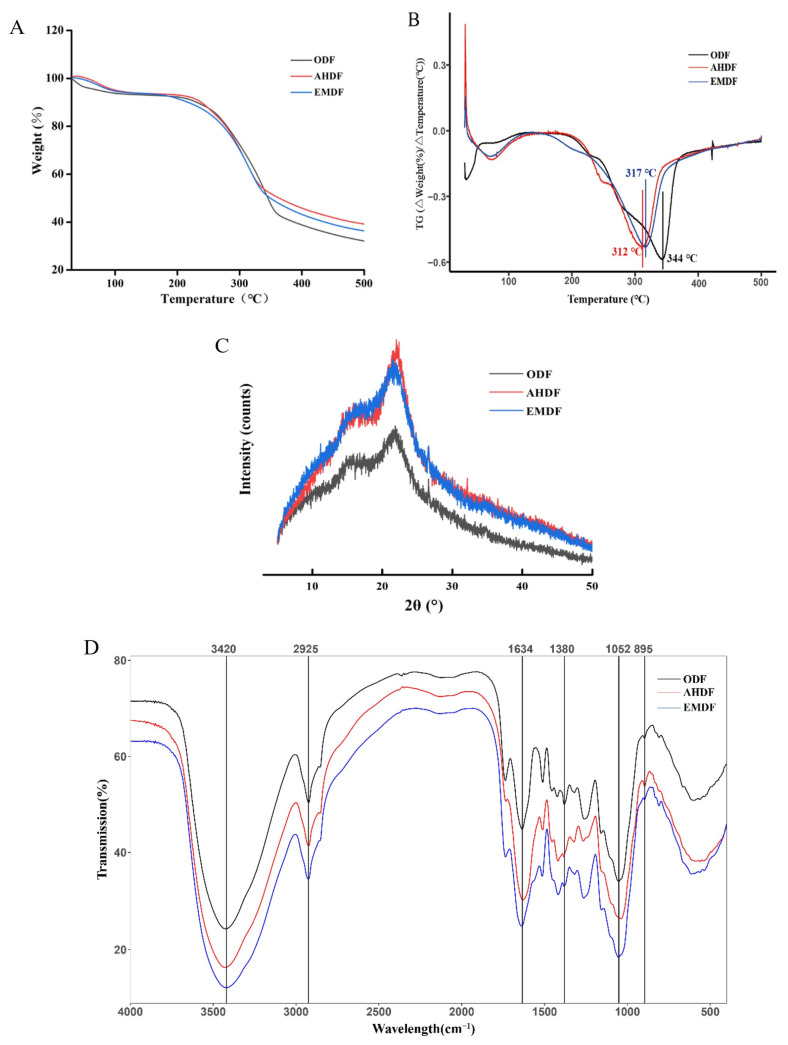
Thermogravimetric analysis (**A**,**B**), X-ray diffraction pattern (**C**), and FT-IR spectrum (**D**) of *Forsythia suspensa* DF before and after modification.

The thermal stability of the DF was measured using TGA [2]. Three phases of thermal decomposition of the samples could be observed (Figure 2A,B). In the first stage (30–200 °C), the weight loss was approximately 10% during this process, and the evaporation of water molecules in the DF was the main reason for the mass reduction [2]. Subsequently, in the second pyrolysis stage (200–320 °C), the rapid decrease in DF mass was due to the dissociation of hydrogen bonds and polysaccharide chains [2]. At 200 °C, the residual masses in AHDF (91.23%) were higher than those in EMDF (89.88%) and ODF (89.91%). However, the residual mass was lower in EMDF and AHDF at 320 ℃ compared to ODF. These results indicated that AHDF and EMDF had higher pyrolysis rates than ODF during the second stage. The thermal stability capacity of DF is reported to be closely related to the bonds between cellulose chains [26]. Similar results were shown in the study by Xie et al. [26], where rice bran DF had a lower residual mass in this stage after modification. When the temperature exceeds 320 °C, DF undergoes the process of biocarbonization [24]. The residual masses of ODF were lower than those of AHDF and EMDF at 500 °C. In contrast, the residual fiber masses of *Mesona chinensis* Benth were higher after modification at the same temperature, which could be related to the types of substances [2].

#### 2.2.3. XRD Analysis

The XRD intensity profiles are shown in Figure 2C. Compared to the unmodified samples, the modified DF samples presented similar typical diffraction peaks of cellulose I. Two characteristic diffraction peaks for all samples were found at 14.00–16.00° (2θ) and 21.40–22.30° (2θ) in the XRD pattern, which were amorphous peaks [2]. This phenomenon was shown in citrus peel DF [24]. Meanwhile, the diffraction peaks of amorphous regions in AHDF and EMDF were steeper and sharper than those in ODF. This could be attributed to the increase in the proportion of the amorphous region in DF after modification [27]. These observations suggest that some crystal regions of *Forsythia suspensa* DF could be destroyed after modification. These phenomena were associated with the loose structure and network composition of the treated DF component.

#### 2.2.4. FT-IR Spectroscopy Analysis

The FT-IR spectrum of the tested samples is shown in Figure 2D. The FT-IR peak at 3000–3700 cm^−1^ is the hydrogen bond region [27]. Compared to ODF, the FT-IR spectrum of AHDF and EMDF had significantly higher response values, which was mainly due to the damage to polysaccharide chains and the greater number of exposed functional groups after modification [28]. Furthermore, the stretching vibrations of C=O and C-O might be the reason for the absorption peaks at 1634 cm^−1^ and 1052–1380 cm^−1^ [29]. The differences in their absorption intensities were associated with the monosaccharide content [2]. Overall, the shapes and sites of the spectrum peaks for all samples were similar, but the absorption intensities of the characteristic absorption peaks showed differences after modification.

### 2.3. WHC, WSA, and OHC Analysis

As shown in Table 2, the WHC and WSA values of AHDF (4.07 g/g and 3.56 mL/g, respectively) were 1.25 and 3.04 times higher than those of ODF. The reason might be that the AHP treatment removed some proteins around the DF and generated a wrinkled structure with many pores (Figure 1B,b), which was convenient for the diffusion of water molecules [30]. The improvement in the WHC and WSA values of AHDF could decrease the dehydration of food products. On the contrary, the WHC and WSA values (2.97 g/g and 0.91 mL/g, respectively) of EMDF decreased compared to those of ODF, which is similar to the observations of Tan et al. [27], who found that the WHC of coconut residue fibers was reduced after modification. Some studies found that the WHC of DF was associated with its degree of polymerization and that extremely high or low degrees of polymerization could significantly reduce the WHC of polymers [27,31]. Thus, the decreased WHC of EMDF could be attributed to low polymerization levels caused by the hydrolysis of cellulase. The cellulase hydrolysis in EMDF could break the molecular structure of the DF, as shown in the SEM images (Figure 1C,c). Therefore, the WSA of EMDF was reduced after the EM treatment.

OHC is regarded as the means to assess the capacity of DF to delay the absorption and digestion of lipids. A high OHC could decrease lipid loss in high-fat foods. As shown in Table 2, the OHC value of AHDF and EMDF increased by 25.6% and 37.3% compared to ODF, respectively. Some reports found that the OHC values of the DF in okara and ginseng were enhanced after APH and EM treatments [23,32], similar to our study. Meanwhile, the OHC of DF samples was associated with their hydrophobic properties and surface characteristics [22]. As mentioned above, the microstructure area of AHDF and EMDF was enhanced after the AHP and EM treatments (Figure 1). Therefore, the increase in the surface area of AHDF and EMDF might be one of the reasons for the improvement in the OHC of *Forsythia suspensa* DF. Furthermore, lipid molecules could diffuse into the DF structure to increase the OHC values when the hydrophobic groups of DFs are exposed after modification.

### 2.4. CEC Analysis

The effects of the two modification methods on the CEC of the DF are shown in Table 2. From Table 2, it is clear that both the AHP and EM modifications could significantly enhance the CEC of *Forsythia suspensa* DF (*p* < 0.05), which may be due to the exposure of ion binding sites, increasing its cation exchange capacity [14]. This is inconsistent with the findings of Wang et al. [14]. Therefore, *Forsythia suspensa* DF after modification could strongly bind to cations and affect the osmotic pressure and pH of the digestive tract, which reduces the risk of disease due to excessive salt intake.

### 2.5. CAC Analysis

**Figure 3:**
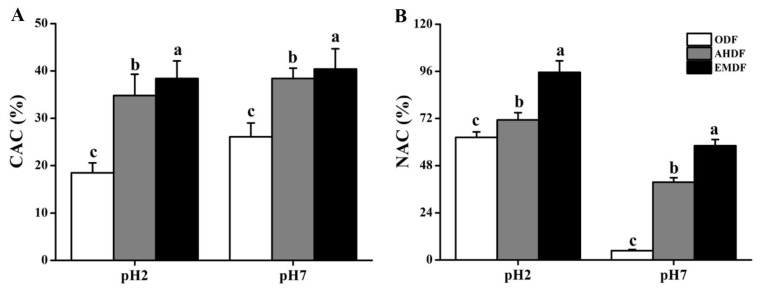
Effects of modification methods on CAC (**A**) and NAC (**B**) of *Forsythia suspensa* DF. Values are expressed as means ± SDs (*n* = 3). Different lowercase letters indicate remarkable differences between groups at *p* < 0.05.

CAC is generally considered an evaluation method to measure the hypolipidemic activity of DF [20]. As shown in Figure 3A, the CAC value in all groups was lower at pH 2.0 than at pH 7.0, indicating that the cholesterol adsorption effects of the DF might be stronger in the gut than in the stomach. When the pH was 7.0, the CAC value was higher for AHDF (38.4%) and EMDF (40.3%) than for ODF (26.1%). Some studies found that the OHC and surface characteristics of DF could affect CAC [33,34]. From the SEM results, both AHDF and EMDF showed an irregular and loose surface structure, which might facilitate easier contact with cholesterol. Furthermore, in this study, from the FT-IR results, some non-polar groups in the DF were exposed after the EM and AHP treatments (Figure 2D), which efficiently enabled AHDF and EMDF to bind with cholesterol. Meanwhile, the looseness of the microstructure of AHDF and EMDF (Figure 1) might be beneficial to improving their cholesterol adsorption capacity. The high OHC of AHDF and EMDF (Table 2) also enhanced cholesterol absorption. Thus, these results suggest that AHDF and EMDF have the potential to reduce hyperlipidemia.

### 2.6. NAC Analysis

Nitrite ions could form N-nitroso substances, which are compounds that are carcinogenic to humans. Thus, the enhancement of NAC might protect against the development of cancer. Figure 3B shows the NAC of the DF before and after modification. The NAC value for all groups was higher at pH 2.0 than at pH 7.0, suggesting that the DF primarily absorbs nitrite ions through the stomach. Compared to ODF, the NAC of AHDF and EMDF increased by approximately 14% and 53% at pH 2, respectively. This suggests that the AHP and EM treatments could improve the NAC of *Forsythia suspensa* DF. Similarly, Qi et al. [19] found that the NAC of the DF of potato residue increased by 26% after modification. Similar to CAC, NAC is associated with the exposure of active groups and the surface area of DF [19,35]. Nitrite ions and functional groups have been reported to form nitrogen oxides to carry out nitrite ion scavenging [1]. In this study, according to the SEM and FT-IR results, the surface area of AHDF and EMDF was enhanced, and some functional groups of AHDF and EMDF were exposed. Therefore, the NAC of *Forsythia suspensa* DF was greatly strengthened after the AHP and EM modifications.

### 2.7. Hypoglycemic Capacity In Vitro

#### 2.7.1. GAC Analysis

DF has the capacity to reduce blood sugar by delaying or reducing glucose absorption [3]. As shown in Table 3, compared to ODF (4.46 μmol/g), the GAC values of AHDF and EMDF were higher, which were 13.93 and 18.94 μmol/g, respectively. Some studies found that the GAC value of DF was related to porous structures with low crystallinity [2,36]. The SEM images in Figure 1 and the XRD pattern in Figure 2 reveal that the AHP and EM treatments generated porous and low-crystallinity structures in AHDF and EMDF, providing more binding sites and increasing the contact area for glucose. Thus, these factors resulted in a relatively higher GAC for AHDF and EMDF. The above results indicated that the glucose absorption capacity of the DF was increased after treatment with APH and EM, which is useful for decreasing the glucose content in the blood.

#### 2.7.2. GDRI Analysis

The GDRI is used to assess the delay in glucose diffusion in DF [3]. As shown in Table 3, the GDRI of AHDF and EMDF was 33.3% and 27.8%, respectively, which was higher than that of ODF (17.9%). Similarly, Tang et al. [3] found that the GDRI of the DF of bamboo shoots was significantly improved after modification. Some studies confirmed that the functional groups in DF have a strong affinity for glucose, which is beneficial in reducing glucose diffusion [37,38]. In addition, the GDRI is related to functional groups and DF surface structures [38]. Some studies confirmed that functional groups, such as aldehydes, have a strong affinity for glucose and might reduce the diffusion of glucose [37]. Combined with the results in Figure 1 and Figure 2, it appeared that AHDF and EMDF had a high GDRI, which could be attributed to the porous surface structure and the exposure of more aldehyde groups after modification. These results indicate that AHDF and EMDF have an important role in decreasing the glucose concentration in the digestive tract.

#### 2.7.3. α-Amylase Inhibitory Activity Analysis

The activity of α-amylase is used to evaluate the rate of gluconeogenesis [2]. Therefore, inhibitory activity against α-amylase could be a means of managing type 2 diabetes by decreasing the conversion of glucose in the blood [39]. The α-amylase inhibition activity of the DF is presented in Table 3. Compared to ODF, the inhibition effects of AHDF and EMDF against α-amylase activity increased by 0.43 and 2.26 times, respectively. Similarly, the α-amylase inhibitory activities were enhanced after modification [32]. Some studies found that the loose structure and large surface area of DF reduced the possibility of starch and enzymes reacting, which could slow the rate of glucose release [10,32]. Additionally, GAC also affected the α-amylase inhibition activity of DF, which was associated with a reduction in glucose diffusion and an improvement in competition with food carbohydrates [1]. As observed above in Figure 1 and Table 3, the porous structure and high GAC allowed AHDF and EMDF to present strong inhibitory activity against α-amylase. Thus, *Forsythia suspensa* DF after AHP and EM treatments has great potential to control type 2 diabetes.

### 2.8. Antioxidant Activities In Vitro

#### 2.8.1. TPC and TFC

DF is an important carrier of polyphenols and flavonoid substances. Therefore, the content of polyphenols and flavonoids in the DF was measured. As shown in Table 4, the TPC value of AHDF and EMDF was 0.30 mg GAE/g and 0.28 mg GAE/g, respectively, which showed no significant differences from that of ODF (0.28 mg GAE/g). This result indicated that the TPC content of the DF did not experience a remarkable change after the modification, similar to research on rice bran DF after modification [40]. In contrast, the TFC concentrations in AHDF and EMDF decreased compared to ODF. Similar reductions in TFC were observed in a report on wheat straw DF after modification [10]. Jiang et al. [36] also found that the TFC value of ginseng DF was decreased by 25% after modification. Some studies revealed that phenolics are covalently linked to polysaccharides in the plant cell wall, which could form strong ester bonds [41], whereas flavonoids might remain in the cytosol in free form [41]. The reason for the lower TFC in AHDF and EMDF might be that flavonoids in the matrix are more likely to be released and exposed after being treated with AHP and EM. On the contrary, the ester bonds of phenolics might not be easily destroyed, explaining why the TPC content in AHDF and EMDF showed no remarkable change. These observations indicate that the AHP and EM treatments of the DF had less effect on the TPC content than on the TFC value.

#### 2.8.2. Radical Scavenging Capacity and FRAP

The antioxidant activities of DF are assessed through the capacity to scavenge radicals or reduce metals. As shown in Table 4, compared to ODF, the DPPH radical scavenging capacities of AHDF and EMDF increased by approximately 29% and 20%, respectively. This observation suggests that the DPPH radical scavenging capacity of DF could be improved after APH and EM modifications. However, the ABTS radical scavenging capacities of AHDF and EMDF were lower than those of ODF, which decreased by about 37% and 14% (Table 4), respectively.

As shown in Table 4, the FRAP value of ODF was 6.32 μmol Fe(II)/g, while the FRAP value was 3.35 μmol Fe(II)/g and 4.84 μmol Fe(II)/g for AHDF and EMDF, respectively. In this study, the FARP and ABTS values of the DF decreased after the APH and EM modifications, especially for AHDF, which showed the greatest reduction. The decreased ABTS scavenging capacity of AHDF and EMDF might be attributed to the decrease in their TFC.

## 3. Materials and Methods

### 3.1. Materials

*Forsythia suspensa* fruits, which were harvested in the region of Anze County, Shanxi Province, China, in 2020, were obtained as commercial products from a local market in March 2021.

### 3.2. Extraction of Forsythia Suspensa DF

*Forsythia suspensa* fruits were crushed, passed through 60-mesh sieves, and then extracted with 70% ethanol for 6 h. After filtration, the residue was successively defatted, lyophilized at 30 °C, and passed through 80-mesh sieves. The defatted *Forsythia suspensa* was added to deionized water, and the pH was maintained at 7.0. Thereafter, 2% mixed enzyme (α-amylase–glycosylase = 1:1) was added and shaken at 60 °C for 1 h, and then 1.2% protease was added to the mixture, which was reacted at 37 °C for 90 min. *Forsythia suspensa* DF (ODF) was then obtained via filtration, washed to neutral with distilled water, centrifuged, lyophilized at 30 °C, and stored at −4 °C until further use. After analysis, ODF contained 0.1% soluble dietary fiber (SDF) and 87.1% insoluble dietary fiber (IDF).

### 3.3. Modification of Forsythia Suspensa DF

The AHP modification was based on a previous research method with slight changes [11]. According to the ratio (1:20 (*m*/*v*)) of ODF to solution, ODF and 1% hydrogen peroxide were mixed to react at 60 °C for 4 h. The pH was then adjusted to 6.0 with 1 mol/L HCl, followed by standing and layering at room temperature. Then, 4 times anhydrous ethanol was added to the supernatant and centrifuged at 6000× *g* for 10 min. Finally, the precipitate was lyophilized at 30 °C to generate AHP-modified *Forsythia suspensa* DF (AHDF). After analysis, AHDF contained 14.5% soluble dietary fiber (SDF) and 69.2% insoluble dietary fiber (IDF).

The EM modification was based on an earlier research method with some changes [42]. According to the ratio (1:20 (*m*/*v*)) of ODF to solution, ODF was added to 0.4% cellulase (pH 4.8), and the mixtures were stirred and incubated at 50 °C for 1 h. The resulting mixtures were heated at 100 °C for 30 min to terminate the enzymatic reaction and allowed to cool to room temperature. After layering, 4 times anhydrous ethanol was added to the supernatant and centrifuged at 6000× *g* for 10 min. The precipitate was lyophilized at 30 °C to generate EM-modified *Forsythia suspensa* DF (EMDF). After analysis, EMDF contained 3.1% soluble dietary fiber (SDF) and 82.3% insoluble dietary fiber (IDF).

### 3.4. Chromaticity

The color difference values of the DF powders were determined using an NS800 spectrophotometer. Color properties were expressed as L*, a*, and b*. The color difference was assessed using the following equation:

$$\Delta E=\sqrt{{\left(L-L_{0}\right)}^{2}+{\left(a-a_{0}\right)}^{2}+{\left(b-b_{0}\right)}^{2}}$$

where *L*_0_, *a*_0_, and *b*_0_ are the chromaticity values of the ODF powder, and *L*, *a*, and *b* are the chromaticity values of the modified DF.

### 3.5. Scanning Electron Microscopy (SEM)

The dried DF samples were fixed on a metal holder, coated with gold via ion sputtering, and then placed under a scanning electron microscope (JSM-7500F, JEOL Ltd., Tokyo, Japan) operated at an acceleration voltage of 5–10 kV to observe the apparent structures of the DF samples.

### 3.6. Thermogravimetric Analysis (TGA)

The DF samples were placed on a thermogravimetric analyzer (TGA/DSC 1/1600HT, METTLER-TOLEDO, Greifensee, Switzerland) to measure the thermostabilities. The conditions were as follows: temperature range, 30–500 °C; nitrogen gas stream, 50 mL/min.

### 3.7. X-Ray Diffraction (XRD)

The DF samples were placed into a sample holder and examined using X-ray diffraction (Ultima IV-185, Rigaku, Tokyo, Japan). The samples were scanned from 5° to 50° (2θ) at 5°/min, and the working voltage and current were 40 kV and 30 mA, respectively.

### 3.8. Fourier-Transform Infrared (FT-IR) Spectroscopy

An amount of 2 mg of DF sample was added to approximately 0.1 g of KBr powder and pressed to generate thick tablets of 1–2 mm. The organic functional groups of the samples were analyzed using an FT-IR spectrophotometer (Woke, Tianjin, China). The wavelength spectrum was between 500 cm^−1^ and 4000 cm^−1^, with a resolution of 4 cm^−1^.

### 3.9. Hydration Properties

The WHC was determined according to the following methods. The sample (1.0 g) was added to deionized water (40 mL) and maintained at 37 °C for 24 h. Then, the precipitate was obtained after centrifugation at 6000× *g* for 15 min. The WHC was assessed according to the equation WHC (g/g) = (M_2_ − M_1_)/M_1_, where M_1_ is the weight of the dry DF, and M_2_ is the weight of the DF containing water.

The OHC was determined according to the following methods. The sample (1.0 g) was placed in a centrifuge tube with soybean oil (20 mL), which was then placed in a thermostatic oscillator at 37 °C for 24 h. The supernatant was removed after centrifugation at 4000× *g* for 20 min. The OHC was measured as follows: OHC (g/g) =(M_2_ − M_1_ − M_0_)/M_1_, where M_0_ is the weight of the centrifuge tube, M_1_ is the weight of the dry DF, and M_2_ is the weight of the DF containing oil and the centrifuge tube.

The WSA was determined according to the following methods. One gram of sample (M) was placed in a measuring tube, and its volume was recorded as V_1_. The sample–deionized water mixture was maintained at 37 °C for 24 h. The volume of the samples after expansion was expressed as V_2_. The WSA was assessed according to the following equation: WSA (mL/g) = (V_2_ − V_1_)/M.

### 3.10. Cation Exchange Capacity (CEC)

The CEC was determined by referring to the method described by Zheng et al. [35] with slight modifications. The DF (0.5 g) was suspended in 50 mL of 0.1 mol/L HCl, mixed well, and maintained at room temperature for 24 h for acidification. After filtration, the precipitate was washed until no precipitate was produced after adding a drop of AgNO_3_ with a concentration of 20%, and then dried at 50 °C for 12 h. The acidic DF was suspended in distilled water, followed by the addition of 2–3 drops of phenolphthalein, and then titrated with 25 mmol/L NaOH. The CEC was expressed as the number of milliequivalents per gram of dry sample (mmol/g).

### 3.11. Cholesterol Adsorption Capacity (CAC)

The measurement of CAC was based on a previous research method with some modifications [37]. Deionized water was added to fresh egg yolks, forming an emulsion after being stirred. Then, the DF sample (1.0 g) and the diluted egg yolk solution (25 mL) were mixed. The pH of the tested samples was maintained at 2.0 and 7.0. The samples were stirred at 37 °C for 2 h, and the supernatant was obtained after centrifugation at 4000× *g* for 20 min. The optical density of the supernatant was analyzed at 550 nm, and the cholesterol concentrations were assessed using the standard curve: y = 10.78x + 0.0006 (R^2^ = 0.9997). The concentrations of cholesterol and CAC were determined according to the ophthalaldehyde method.

### 3.12. Nitrite Adsorption Capacity (NAC)

The NAC was measured according to a previous research method [1]. The DF sample (1.0 g) was added to sodium nitrite solution (25 mL, 100 μmol/L). The pH value of the mixture was maintained at 2.0 and 7.0. Then, the sample was reacted at 37 °C for 120 min. Nitrite concentrations were measured using N-(1-Naphthyl) ethylenediamine dihydro-chloride/sulfanilic acid solution at an absorbance of 538 nm.

### 3.13. Glucose Adsorption Capacity (GAC)

The GAC was assessed according to the method of Tang et al. [3]. The DF sample (0.5 g) was mixed with glucose solution (2.0 g/L). Then, the levels of glucose were measured using a glucose kit. The GAC was expressed as mmol of retained glucose per g of DF.

### 3.14. Glucose Dialysis Retardation Index (GDRI)

The sample (0.5 g) and glucose solution (2.0 g/L) were placed in a dialysis membrane (3000 Da). Then, the dialysis membrane was placed into a container with 200 mL of deionized water and stirred at 37 °C. The blank control (without dietary fiber and with glucose) and control sample (with dietary fiber and without glucose) were obtained, and 20 μL of dialysis solution was collected. The GDRI was assessed according to the following formula:

$$GDRI(\%)=\frac{[1-(C-C_{d})]100}{C_{0}}$$

where C is the glucose concentration of the sample solution (μg/mL); C_d_ is the glucose concentration of the control sample (μg/mL); and C_0_ is the glucose mass concentration of the blank control (μg/mL).

### 3.15. α-Amylase Inhibitory Activity (AIA)

The inhibitory activity of α-amylase was measured according to the method of Yang et al. [2] with slight changes. The DF (1.0 g) and 5 mg/mL of soluble starch solution were mixed. Then, α-amylase (40 U/mg) was added to the mixture, and the pH was maintained at 6.5. Finally, glucose in the supernatant was obtained after centrifugation at 4000× *g* for 10 min, concentrations of which were assessed using the anthrone colorimetric method. The inhibitory activity of α-amylase was analyzed as follows: α-amylase inhibitory activity (%) = 100 × (Ac − As)/Ac, where Ac is the glucose content in the control, and As is the glucose content in the DF.

### 3.16. Total Phenolic Content (TPC)

The DF samples were mixed with 80% methanol and then centrifuged at 5000× *g* for 15 min. The supernatants were dried at 40 °C and kept at −4 °C for further measurement. The TPC was measured according to the method reported by Yang et al. [2]. Gallic acid (GA) was used to calibrate the standard curve, and the TPC was expressed as milligrams of GA equivalent per g of DF (mg GAE/g).

### 3.17. Total Flavonoid Content (TFC)

The TFC was assessed using the method of Yang et al. [2]. *Forsythia suspensa* extract (1.0 mL) was placed into a test tube, where 5% sodium nitrite solution was added. Then, it was mixed with 4% sodium hydroxide solution to react, and the optical density was analyzed at 510 nm. Rutin was used to calibrate the standard curve, and the amount of flavonoids was expressed as milligrams of rutin equivalent per g DF (mg RE/g).

### 3.18. Antioxidant Activity

The DPPH and ABTS radical scavenging capacities were analyzed according to the method of Thaipong et al. [43]. Different concentrations of Trolox (100–1000 μg/mL) were utilized to establish the standard curve. The result was expressed as micrograms of Trolox equivalents per g of sample (μg TE/g). The ferric reducing antioxidant power (FRAP) assay was carried out based on the method of Yang et al. [2]. FeSO_4_ solution was utilized to establish the standard curve. The FRAP was expressed as micromoles of Fe (II) per g sample (μmol Fe (II)/g).

### 3.19. Statistical Analysis

All values are presented as the mean ± standard deviation (SD). One-way analysis of variance was conducted via SPSS 18.0 (Chicago, IL, USA). The differences were assessed through Duncan’s test and considered significant when *p* < 0.05.

## 4. Conclusions

In this study, the effects of AHP and EM modifications on the physicochemical and functional capacities of *Forsythia suspensa* DF were investigated. Compared to ODF, AHDF and EMDF showed a loose and porous surface microstructure and a low crystallization level after the AHP and EM treatments. In addition, the physicochemical properties such as OHC and CEC of AHDF and EMDF were significantly improved. Moreover, the AHP and EM treatments improved some functional properties including the CAC, NAC, DPPH radical scavenging activity, GAC, GDRI, and α-amylase inhibitory activity. Thus, AHDF and EMDF could be regarded as additives in functional food. APH and EM modification, as a promising technique, could generate high-bioactivity *Forsythia suspensa* DF. These findings contribute to clarifying the relationship between modification and function in *Forsythia suspensa* DF, which will provide a theoretical basis for further applications of *Forsythia suspensa* DF.

## Figures and Tables

**Table 1:** Effect of modification methods on the color of *Forsythia suspensa* DF.

	a	b	L	ΔE
ODF	−1.22 ± 0.13 ^a^	−2.09 ± 0.17 ^a^	54.99 ± 0.24 ^a^	—
AHDF	−1.79 ± 0.25 ^b^	−3.05 ± 0.08 ^b^	55.08 ± 0.19 ^a^	1.13 ± 0.11 ^a^
EMDF	−1.87 ± 0.09 ^b^	−3.22 ± 0.12 ^b^	54.88 ± 0.25 ^a^	1.31 ± 0.14 ^a^

Each value is expressed as the mean ± SD (*n* = three). Different lowercase letters indicate remarkable differences between groups at *p* < 0.05. ODF, unmodified *Forsythia suspensa* DF; AHDF, *Forsythia suspensa* DF treated using alkaline hydrogen peroxide; EMDF, *Forsythia suspensa* DF treated using cellulase.

**Table 2:** Effect of modification methods on hydration properties and CEC of *Forsythia suspensa* DF.

	WHC (g/g)	OHC (g/g)	WSA (mL/g)	CEC (mmol/g)
ODF	3.25 ± 0.0 ^b^	3.24 ± 0.25 ^b^	1.17 ± 0.11 ^b^	0.08 ± 0.02 ^b^
AHDF	4.07 ± 0.05 ^a^	4.07 ± 0.37 ^a^	3.56 ± 0.11 ^a^	0.15 ± 0.03 ^a^
EMDF	2.97 ± 0.02 ^c^	4.45 ± 0.59 ^a^	0.91 ± 0.17 ^b^	0.17 ± 0.05 ^a^

Values are expressed as means ± SDs (*n* = 3). Different lowercase letters indicate remarkable differences between groups at *p* < 0.05.

**Table 3:** Effect of modification methods on GAC, GDRI, and α-amylase inhibitory activity (AIA) of *Forsythia suspensa* DF.

	GAC (μmol/g)	GDRI (%)	AIA (%)
ODF	4.46 ± 0.56 ^c^	17.9 ± 2.9 ^c^	11.5 ± 2.1 ^c^
AHDF	13.93 ± 2.35 ^b^	33.3 ± 3.6 ^a^	16.5 ± 3.1 ^b^
EMDF	18.28 ± 1.14 ^a^	27.8 ± 4.3 ^b^	37.5 ± 0.9 ^a^

Values are expressed as means ± SDs (*n* = 3). Different lowercase letters indicate remarkable differences between groups at *p* < 0.05.

**Table 4:** TPC, TFC, and antioxidant activities of *Forsythia suspensa* DF before and after modification.

	TPC (mgGAE/g)	TFC (mgRE/g)	DPPH (μgTE/g)	ABTS (μgTE/g)	FRAP (μmoL Fe (II)/g)
ODF	0.28 ± 0.02 ^a^	1.25 ± 0.08 ^a^	3.39 ± 0.25 ^c^	43.42 ± 3.05 ^a^	6.32 ± 0.54 ^a^
AHDF	0.30 ± 0.02 ^a^	0.65 ± 0.01 ^c^	4.36 ± 0.36 ^a^	27.07 ± 2.54 ^c^	3.35 ± 0.16 ^c^
EMDF	0.28 ± 0.03 ^a^	1.13 ± 0.02 ^b^	4.08 ± 0.27 ^b^	37.25 ± 1.02 ^b^	4.84 ± 0.25 ^b^

Values are expressed as means ± SDs (*n* = 3). Different lowercase letters indicate remarkable differences between groups at *p* < 0.05.

## Data Availability

Data will be made available upon reasonable request.
